# Supplementary material for: Chiral symmetry breaking yields the I-Au60 perfect golden shell of singular rigidity
Source: Nat Commun. 2018 Aug 22;9:3352. doi: 10.1038/s41467-018-05215-3 (PMC6105599; doi:10.1038/s41467-018-05215-3)
Supplement: Supplementary file 1 — Supplementary Information [file 41467_2018_5215_MOESM1_ESM.pdf]

Supplementary Information:

Chiral Symmetry Breaking Yields the *I*-Au_{60} Perfect Golden Shell of Singular Rigidity

X. López-Lozano et al.

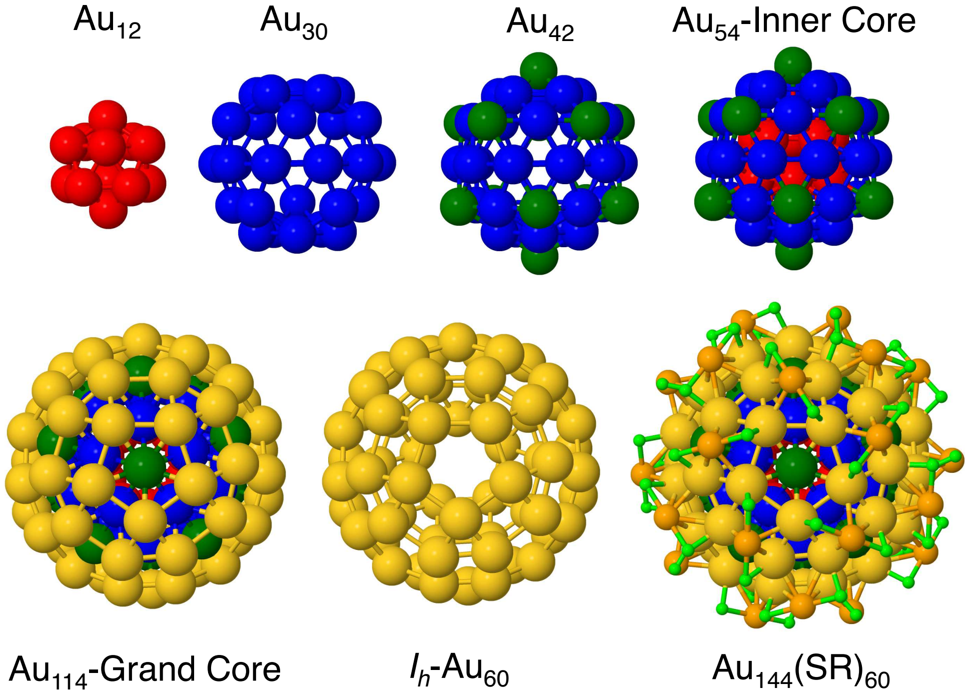

Supplementary Figure 1. Construction of the ubiquitous icosahedral cluster-compound of $Au_{144}(SR)_{60}$. The $Au_{144}(SR)_{60}$ may be decomposed into the so-called Inner Core, Grand Core, and 30 staple motifs. The Inner Core, Au_{54}, comprises a 12-atom shell (red) and a (30 + 12)-atom shell (blue & green). Surrounding the Inner Core is the I_h–Au_{60} surface shell (yellow), giving us the Grand Core of Au_{114}. Finally the $Au_{144}(SR)_{60}$ is obtained by decorating the Grand Core with the 30 staple motifs (RS-Au(I)-SR), (colors gold and lime).[1-5]

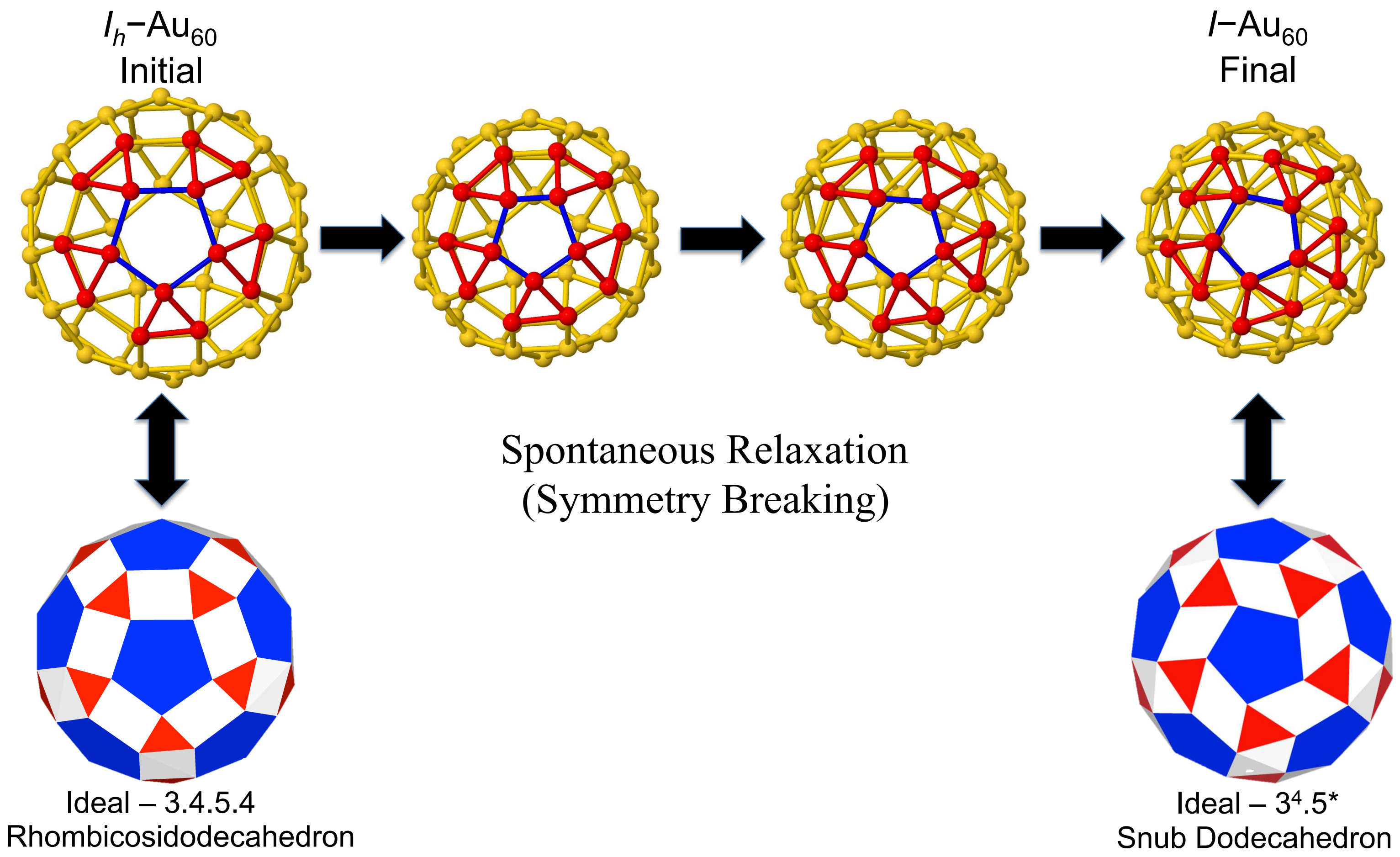

Supplementary Figure 2. Snapshots taken during the spontaneous relaxation of the initial I_h–Au_{60} structure into the I–Au_{60} final structure. This transition is accompanied by a loss of the reflection and inversion symmetries, which occurs during the internal rotation phase. The animation of this structural relaxation can be found in Supplementary Movie 1.

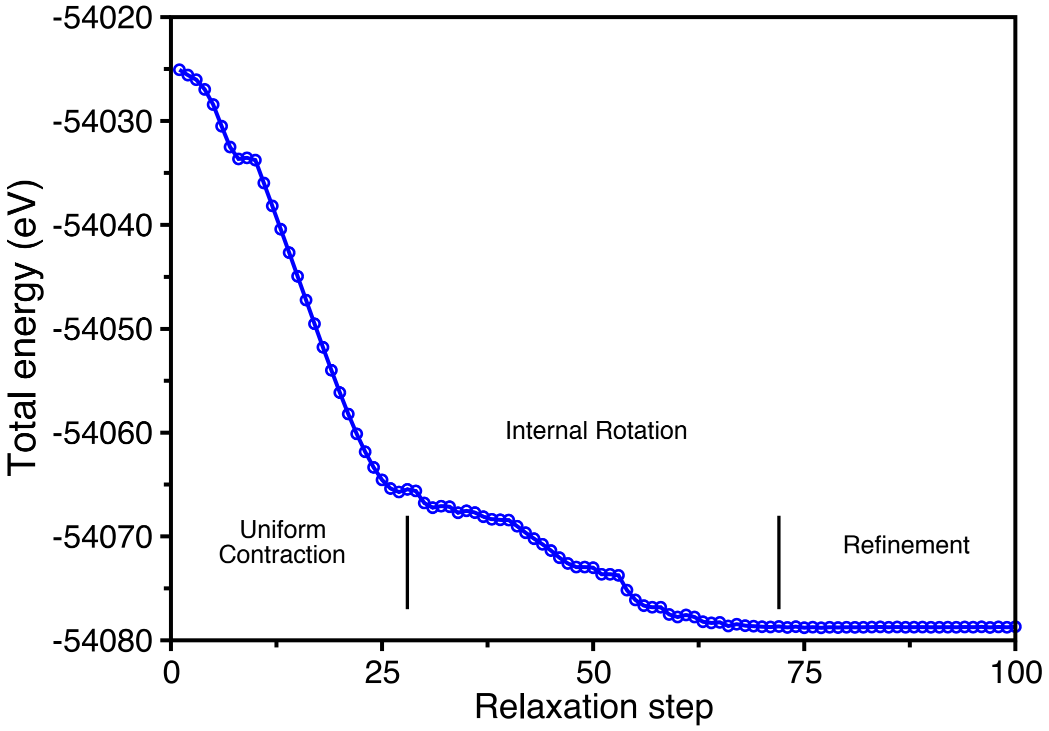

Supplementary Figure 3. Evolution of the total energy of the I_h–Au_{60} structure, obtained at each step during relaxation of the I_h–Au_{60} shell. The structure first undergoes a uniform contraction, indicated by the first vertical line. The majority of the reduction in total energy during the relaxation (~40 eV) comes during this initial contraction. Following the contraction is a rotation of the pentagons associated with the transition from the I_h–Au_{60} rhombicosidodecahedron (3.4.5.4) to the I–Au_{60} snub dodecahedron (3^4.5*) morphology, between the first and second vertical lines. Lastly, there are structural refinements until the relaxation tolerances are met. Atomic coordinates of the initial I_h–Au_{60} and final I–Au_{60} are provided in Supplementary Data 1 and 2.

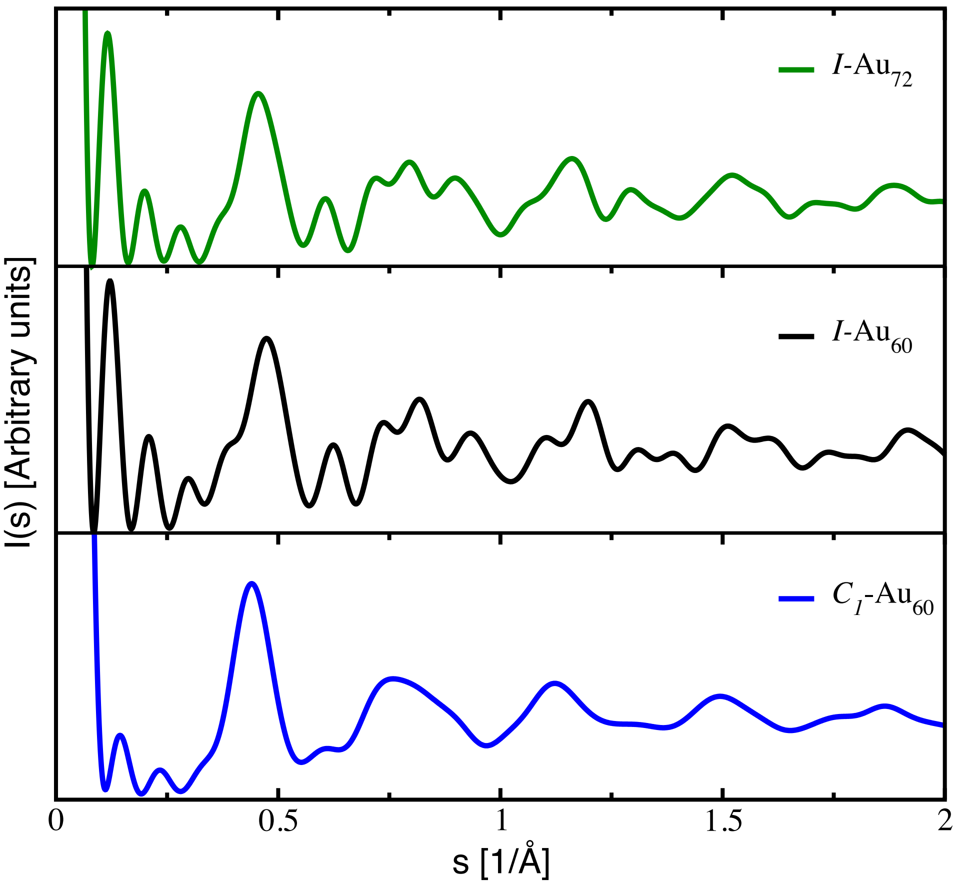

Supplementary Figure 4. Structure Factors of the *I*–Au_{72}, *I*–Au_{60}, and C_1–Au_{60}. The Structure Factors, I(s) vs. s, are calculated from the respective coordinate-sets of each of the indicated optimized gold clusters. These functions are crucial for comparison with results from X-ray or electron scattering (diffraction) measurements. For the compact C_1–Au_{60} structure, cf. Fig. 1(h), the 3D-ordering is reflected in a pattern of maxima that resemble the Bragg reflections of the regular bulk lattice (FCC Au); e.g. the maximum near 0.43 $Å^{-1}$ relates to the stacking of (111)-type lattice planes (2.35 Å spacing). For the chiral-icosahedral shells, *I*–Au_{60} & *I*–Au_{72}, the structure-factor I(s) patterns exhibit profound similarities, except for a contraction of the latter (*I*–Au_{72}), due to its larger diameter, and its notable smearing, reflecting the interference produced by placing the extra (12) atoms in sites at the pentagonal "defects". The shape-factor oscillations, in the small-s (low-angle) region, reflect the overall greater size and symmetry of the chiral-icosahedral shells, as compared to the compact (filled) structure, as is evident by comparing the locations of the first three zeroes (shape-factor minima).

Supplementary Note 1.
The results for the adiabatic (A) and vertical (V) electron affinity (EA) are as follows:

AEA (C_1–Au_{60}) = -2.483 eV
AEA (I–Au_{60}) = -3.431 eV

VEA (C_1–Au_{60}) = -2.791 eV
VEA (I–Au_{60}) = -3.615 eV

Thus the addition of a single electron stabilizes by ~ 1.0 eV (0.95 eV) the I–Au_{60} cage, with respect to the C_1–Au_{60} compact form. Extrapolating twelvefold, i.e. for application to I–Au_{60} $^{[12-]}$, this difference would cancel entirely the energetic advantage of the compact form. This result supports our previous conjecture that this structure will likely be obtained in association with 12 counter-cations.

In summary, the free energy difference between I–Au_{60} and C_1–Au_{60} isomers do not provide enough evidence to assert the stability of the I–Au_{60} isomer at finite temperatures. However, the greater electron affinity of the chiral-icosahedral cage points toward its competitive stability of its anionic forms, realized with counter-cations.

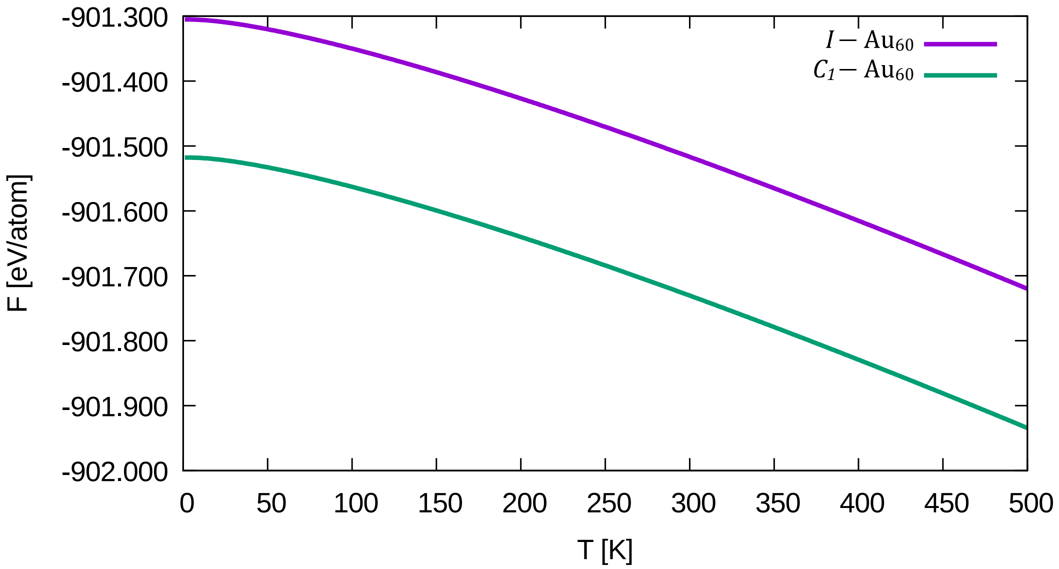

Supplementary Figure 5. To investigate further the stability of the I–Au_{60} we have calculated the electron affinities and Helmholtz free energies of the isomeric I–Au_{60} icosahedral shell and compact C_1–Au_{60} forms. The present figure shows the temperature dependence of the free energy in the harmonic approximation. Although the I–Au_{60} nanocage has a large number of low-frequency modes, a similar behavior is displayed by the C_1–Au_{60} low-symmetry isomer. Therefore, these results do *not* indicate that entropic factors significantly favor the I–Au_{60} isomer. This is consistent with its rigidity, which is unusually high for a quasi-2D (cage) structure.

C_1–Au$_{60}$					I–Au$_{60}$					I–Au$_{72}$				
18.29	55.55	80.98	121.31		17.77	50.16	85.3	136.2		23.07	51.97	71.63	111.12	160.76
22.77	56.01	81.07	122.03		19.92	50.49	86.25	136.88		24.78	52.21	71.88	111.15	160.94
24.44	56.72	81.97	123.58		21.9	50.9	86.27	137.14		26.15	52.49	72.56	111.36	161.07
25.24	57.1	82.58	125.14		23.53	51.38	87.09	137.76		27.81	52.63	75.35	112	161.12
26.26	57.7	83.41	126.54		23.91	51.61	87.39	138.16		27.87	53.19	75.7	112.18	161.51
28.8	58.33	84.06	127.16		25.23	51.85	87.81	143.4		28.87	53.48	75.83	112.6	164.58
30.27	58.54	85.06	127.8		26.83	52.07	88.67	144.44		30.79	54.07	76.32	112.81	164.73
32.1	58.93	86.18	129.47		27.47	52.36	88.79	145.17		31.42	54.35	77.13	112.99	165.13
32.86	59.79	86.77	130.09		27.94	52.92	97.16	146.42		32.22	54.52	78.07	113.36	165.28
34.06	60.23	87.78	131.17		28.58	53.54	97.41	146.58		32.42	54.63	78.63	115.89	165.41
35.01	60.63	89.17	131.7		29.24	53.78	97.68	146.91		33.7	55.13	79.21	116.51	165.69
35.18	61.12	90.32	133.19		29.92	54.03	98.19	147.58		33.86	55.4	79.81	117.06	165.81
36.92	61.42	91.57	134.49		31.35	54.62	98.83	150.04		34.83	55.49	85.07	121.9	166.15
37.17	62.47	92.92	134.97		32.2	54.86	99.93	151.09		35.7	55.85	85.8	122.2	168.73
38.46	62.72	94.03	137.27		32.54	55.46	100.64	151.17		36.22	55.94	86.39	122.33	168.85
38.9	63.05	94.18	137.59		33.98	55.65	101.14	151.59		36.37	56.2	87.46	122.66	169.23
39.71	63.54	95.22	139.22		34	55.85	103.31	157.77		37.15	56.62	88.46	122.79	169.32
40.1	64.37	95.82	139.42		34.83	56.61	104.01	158.05		37.88	56.68	89.23	127.33	169.57
41.45	64.61	96.75	139.81		36	60.31	104.28	158.35		38.14	57.1	89.74	127.58	169.61
42.14	65.42	97.48	142.45		37.07	60.49	110.66	158.4		39.81	57.35	90.49	127.76	169.93
42.49	66.32	98.57	145.11		37.43	61.58	111.85	158.53		40.64	57.37	91.12	128.04	170.28
43.39	66.5	99	146.23		38.37	62.12	112.06	158.81		42.05	58.34	91.31	128.96	171.33
43.67	67.38	100.03	147.06		39.39	62.6	112.49	159.13		42.67	58.73	91.98	129.54	171.4
44.74	67.87	100.14	147.62		39.89	63.08	112.82	159.52		43.36	58.98	92.2	129.95	171.79
45.68	68.08	101.61	149.02		40.15	63.36	113.72	169.03		43.66	59.44	93.26	141.07	171.96
46.15	68.32	102.38	150.26		40.68	65.86	113.89	169.35		43.84	59.99	96.33	141.55	177.24
46.68	68.73	103.19	150.9		41.1	69.41	114.7	169.56		44.91	60.96	96.81	141.71	177.56
47.13	69.42	105.95	151.14		41.68	69.86	124.43	169.84		45.48	61.58	97.12	142.04	178.1
47.44	70.4	106.46	152.58		43.2	70.42	124.54	183.58		45.9	61.89	97.43	142.33	178.21
47.99	70.66	106.57	153.43		43.71	71.13	125.5	183.95		46.69	62.3	97.89	144.42	179.42
48.74	71.07	108.07	153.76		44.18	74.51	125.86	184.18		46.85	62.97	98.3	144.59	179.56
49.16	71.62	108.47	155.15		44.6	74.98	127.46	184.5		47.15	63.2	99.06	144.8	179.79
49.56	72.11	109.58	155.93		44.72	75.53	127.57	184.67		47.37	63.48	99.32	144.87	180.03
50.14	72.85	110.69	157.44		45.33	79.14	128.08	185.2		48.73	64.42	99.64	150.89	180.06
50.33	73.45	112.61	159.04		45.91	79.95	128.66	185.45		48.99	64.69	99.75	151.08	180.37
50.86	75.13	113.33	160.15		46.21	80.19	129.91	185.58		49.68	65.24	100.04	151.15	180.66
51.59	76.03	113.74	161.21		46.6	80.56	130.13	185.78		49.86	65.79	100.49	151.29	180.9
52.65	76.52	115.72	161.93		47.26	80.99	130.48	185.9		49.94	67.62	100.65	151.79	180.98
53.08	77.76	116.64	164.13		48.11	81.49	130.79	186.07		50.36	68	100.98	156.85	
53.64	78.11	117.23	166.52		48.81	82.4	131.12	186.68		50.82	68.4	106.12	157.1	
54.4	78.87	117.87	168.19		48.9	82.84	134.3	186.89		51.28	69.65	109.2	157.5	
54.84	79.84	120.08	173.72		49.18	84.11	134.59	187.25		51.58	70.11	109.55	160.36	
55.04	80.17	120.51	175.54		50.02	84.8	134.98	187.38		51.79	70.49	109.79	160.56	
			178					187.6						
			180.83					188.16						

Supplementary Table 1. Vibrational frequencies (in cm^{-1}) for the C_1–Au$_{60}$, I–Au$_{60}$, and I–Au$_{72}$ structures, which are presented as a vDOS in Figure 5 in the main manuscript. This data confirms that the three cluster-structures correspond to true local minima of their potential energy surface, since all frequencies calculated are real and significantly positive.

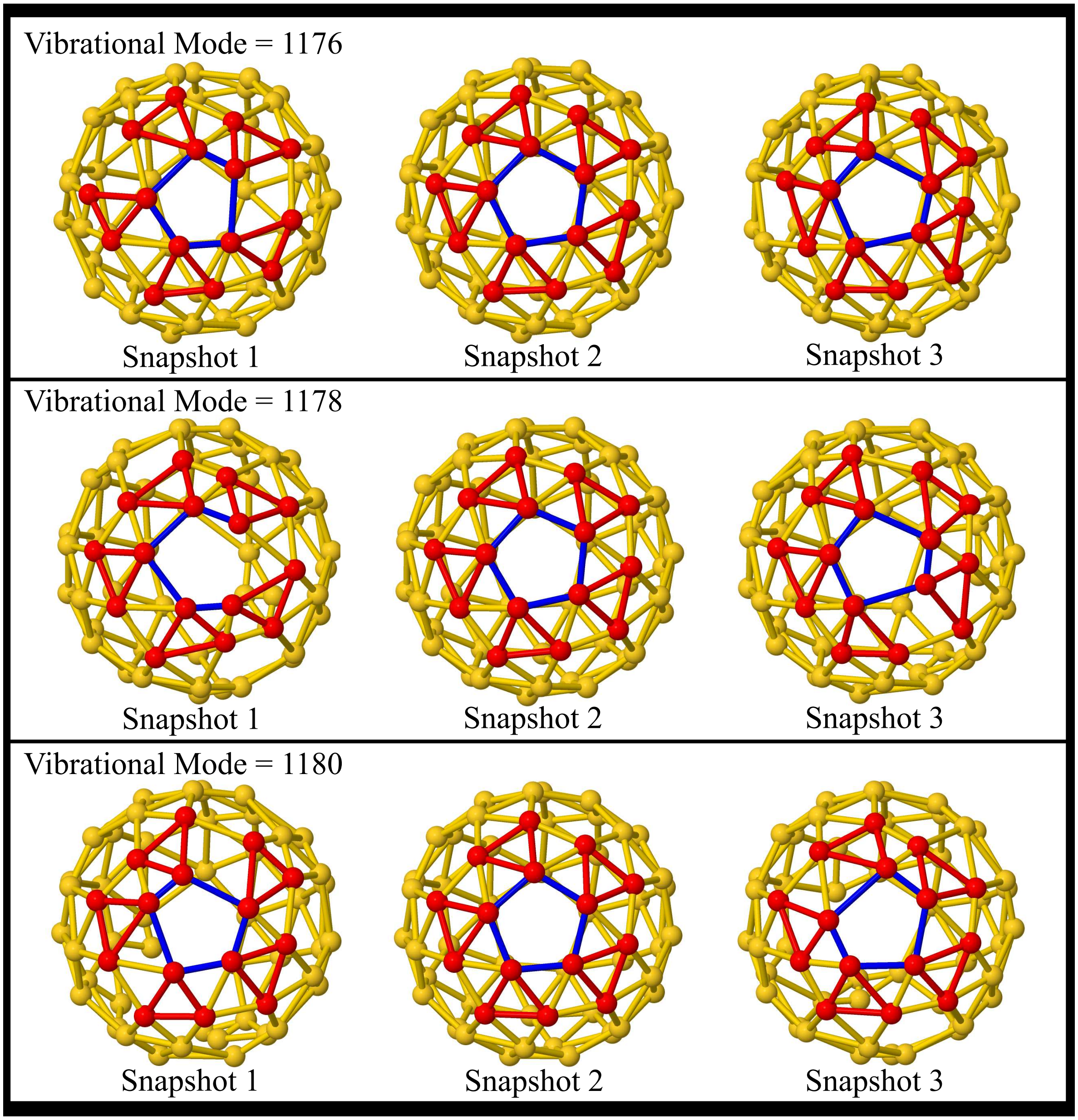

Supplementary Figure 6. Snapshots of selected higher frequency vibrations corresponding to combinations of symmetrical and asymmetrical stretching modes. Animations of the vibrational modes 1176 – 1180 can be found in Supplementary Movies 2 – 6.

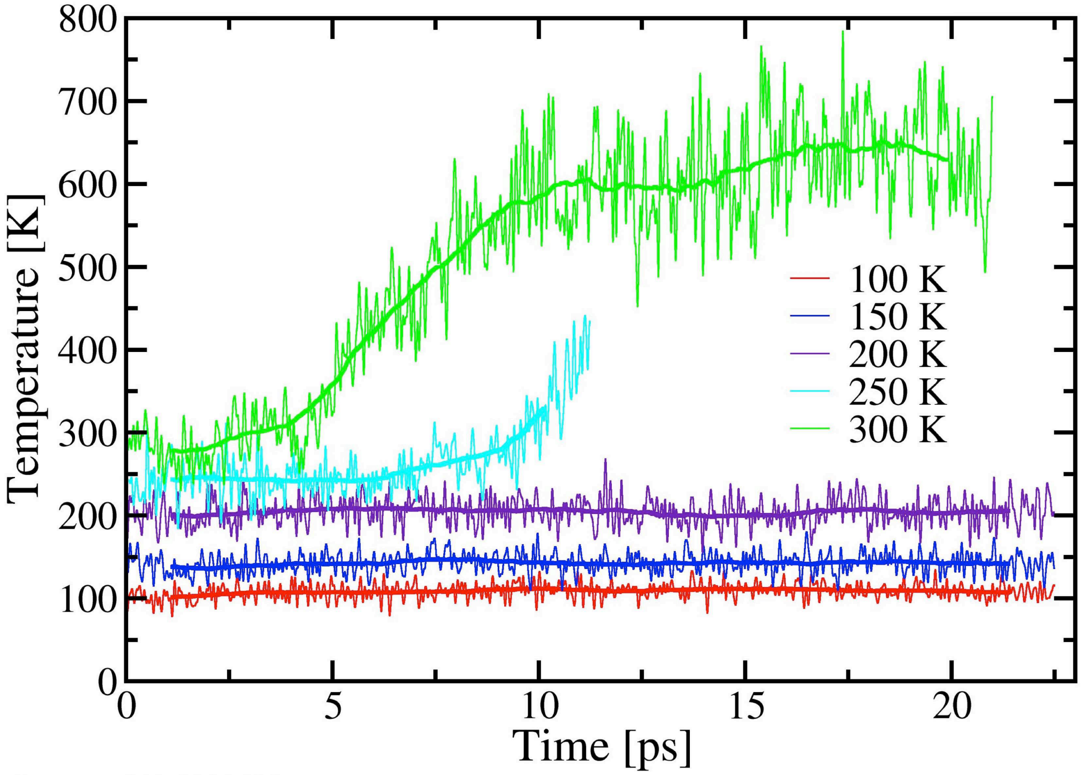

Supplementary Figure 7. The time-dependence of the ionic temperature during the molecular dynamics (MD) simulations in the microcanonical ensemble for the charge-neutral *I*–Au_{60}. The three rather long runs at 100K, 150K, and 200K indicate "stability". For these runs, the temperature is basically constant, i.e., there is no drift or systematic increase of the temperature. By contrast, for 250K and 300K, the temperature rises significantly after about 5 (10) ps. This indicates that the structure has started to move into a lower-energy configuration, changing the connectivity and symmetry. In conclusion, we confirm that the *I*–Au_{60} structure is largely stable up to about 200K but unstable from roughly 250K, see next figure for snapshots of the *I*–Au_{60} structure during the MD calculations.

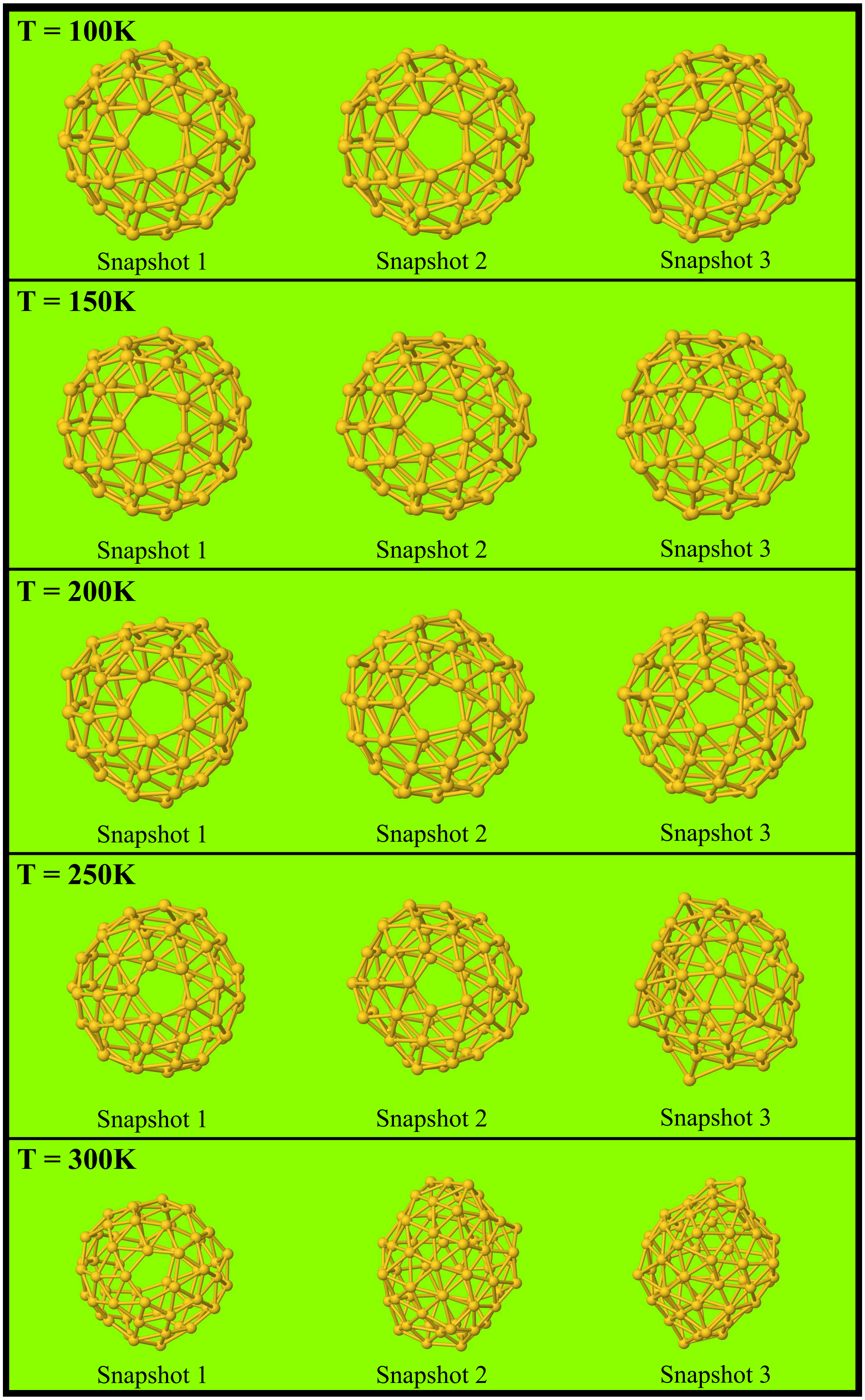

Supplementary Figure 8. Snapshots of the molecular dynamics (MD) calculations at the indicated temperatures. The MD simulations show that at 100K, 150K, and 200K the *I*–Au_{60} cluster retains its cage structure. At 250K, the *I*–Au_{60} isomers become fully distorted and unstable. The MD animations for temperatures of 100K, 150K, 200K, 250K, and 300K can be seen in Supplementary Movies 7–11.

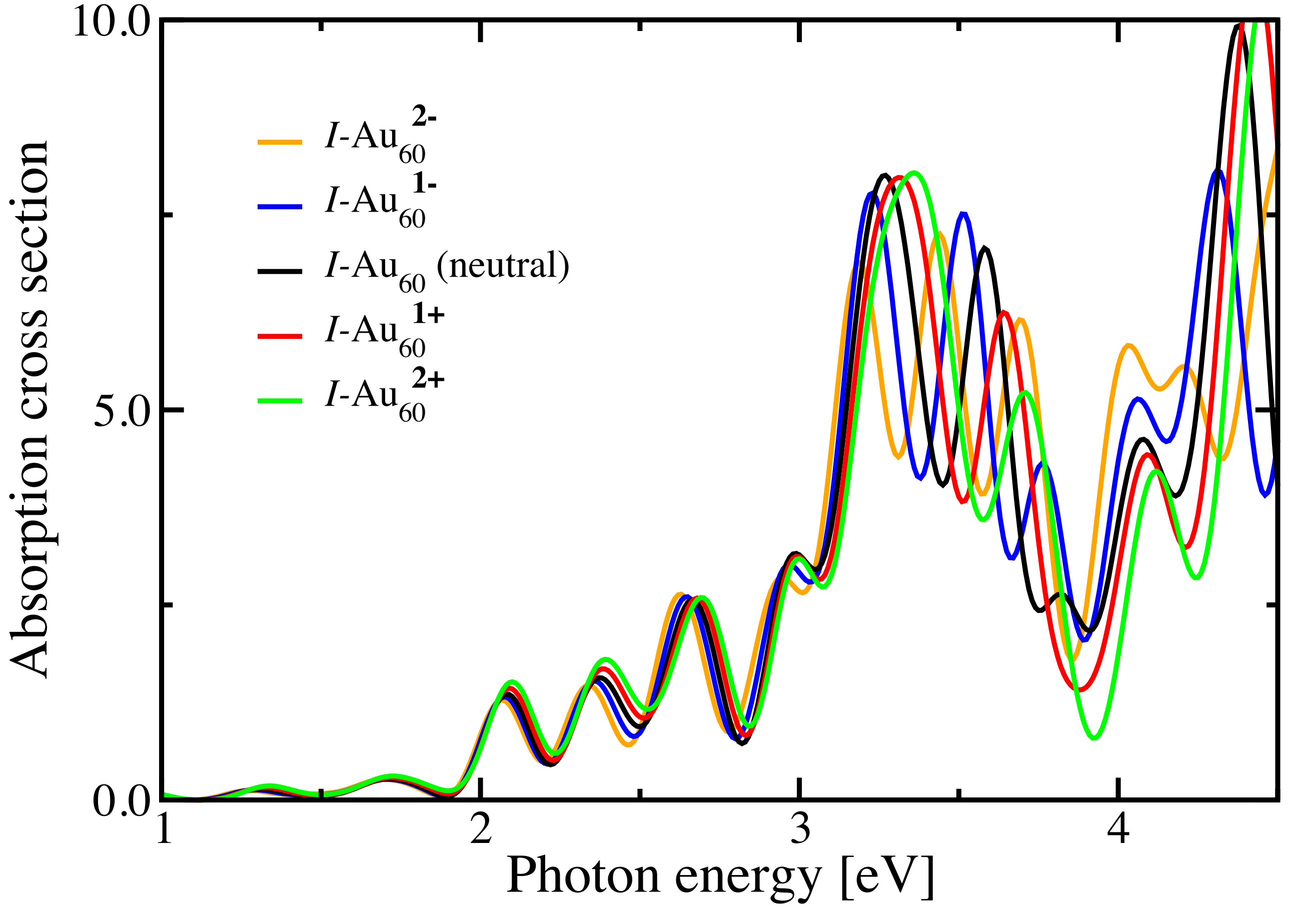

Supplementary Figure 9. Optical absorption spectra of *I*–Au_{60} in different charge states including the neutral form (black). The spectra have been calculated using the PBE functional and the neutral ground-state geometry. The charge-dependence of the spectra in the visible region is rather weak, but is strong above 3 eV.

RT-TDDFT Calculation Shows Modes of Absorption at Different Energies

Excitation along z-direction (i.e., along one of the 5-fold-symmetry axes).

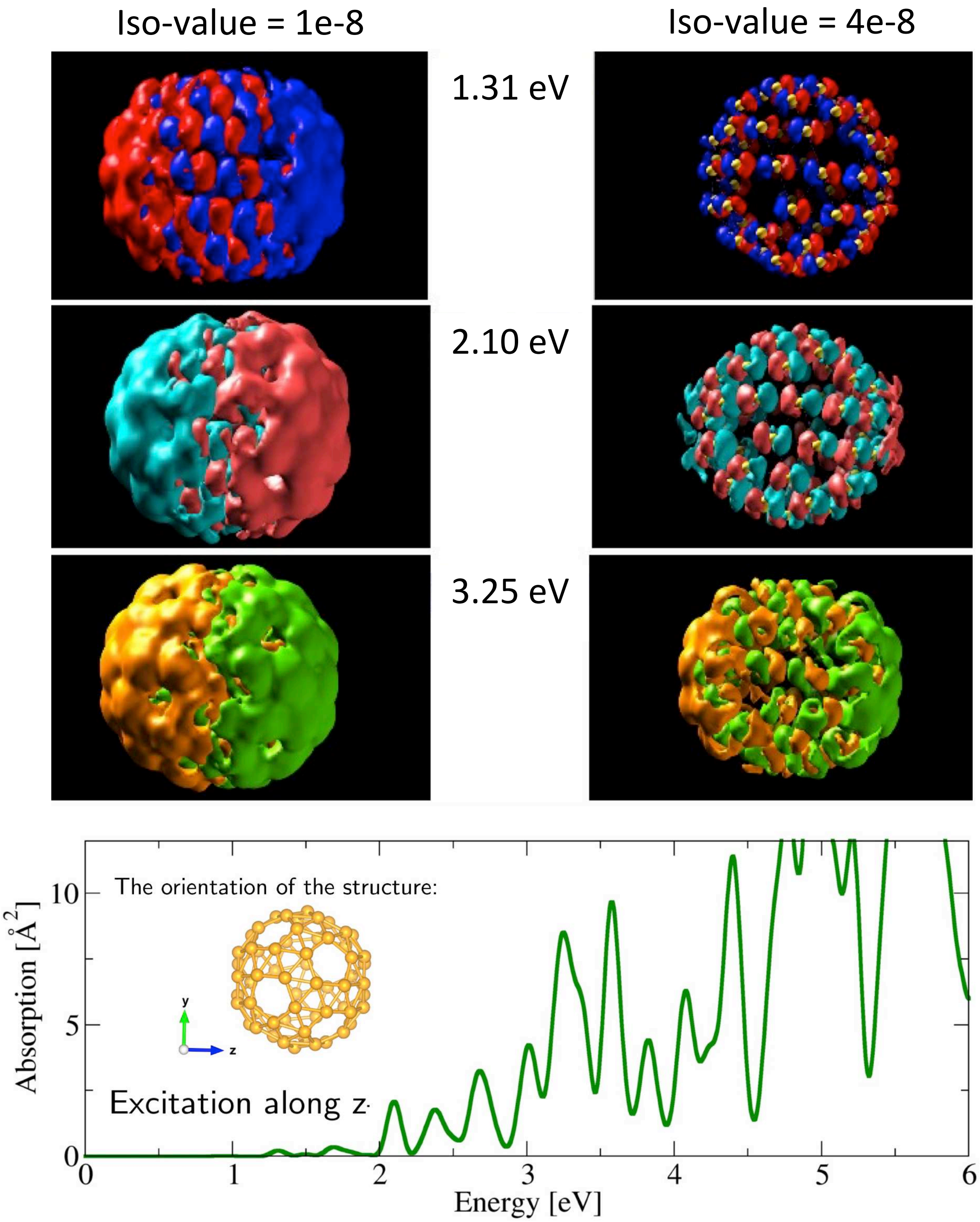

Supplementary Figure 10. We show the induced density corresponding to different modes in the spectrum: 1.31 eV (the very weak peak), 2.10 eV, and 3.25 eV. All isosurfaces are shown for two iso-values as indicated (the same for all three energies.) We can clearly distinguish the usual dipole mode of the delocalized metal *s* electrons and the polarization of the density of the rather strongly bound *d* electrons. The modes are obtained by Fourier transform from a delta-kick calculation.

Supplementary Note 2.
At 1.31 eV, there is an overall dipole mode where the induced density is mostly localized at the facets of the cluster, as seen from the direction of the excitation, along one of the 5-fold symmetry axes (z). This corresponds to the usual surface mode due to the delocalized *s* electrons. Around the "waist" of the shell, this mode has hardly any contributions. Therefore, the polarization of the *d* electrons is visible, more strongly localized around the atoms. This polarization opposes the overall dipole mode. This latter point is even more clearly seen in the figure for higher isovalues, on the right hand side (RHS), where essentially the contributions from the *d* electrons are seen (rather strongly localized and opposed to the overall dipole oscillation of the delocalized *s* electrons). Note that on the left hand side, the screw-like stripes show nicely the chiral nature of the I–Au_{60} shell.

In addition, the amplitude of the overall oscillation of the delocalized *s* electrons is stronger at 2.10 eV than at 1.31 eV, and yet stronger at 3.25 eV (as inferred from the strengths of the corresponding peaks in the spectrum); it is invisible in the high-isovalue figure (RHS) at 1.31 eV, it produces a small contribution in the RHS figure for 2.10 eV, whereas it is strongly visible and mixes entirely with the *d* contribution for the strong resonance at 3.25 eV.

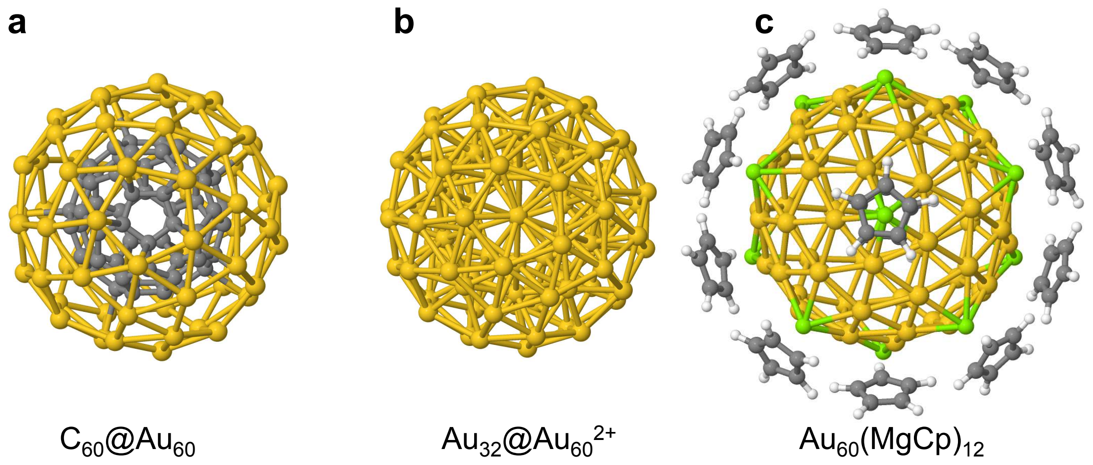

Supplementary Figure 11. Optimized structures of models based on the *I*–Au_{60} system: **(a) I_h–C_{60}@ *I*–Au_{60}:** The I_h–C_{60} fullerene core was placed inside the *I*–Au_{60} and aligned according to the shared icosahedral symmetry. Upon relaxation, the core rotates with respect to the shell, and so the alignment of icosahedral symmetry was lost; however each shell keeps its respective symmetry. The Au_{60} is less spherical with an increase in radius of approximately 4.75% with respect to the isolated *I*–Au_{60} shell, while the Au–Au bond lengths are between 2.82 Å and 2.92 Å. The HOMO-LUMO gap is 0.56 eV.
(b) I_h–Au_{32}@*I*–Au_{60}^{2+} bilayer structure: The core is composed by an I_h–Au_{32} core[6] and the *I*–Au_{60} shell surrounding it. Shell symmetries are maintained upon relaxation. In contrast to the isolated *I*–Au_{60} shell, the Au_{60} shell from the I_h–Au_{32}@*I*–Au_{60}^{2+} structure has a radius of ~6.4 Å, an increase of 8.47%. This is also reflected in the bond lengths with a uniform increase of 12.22% in the pentagons, and 3.28% and 4.66% for the triangles. $Au_{32}@Au_{60}$ has 92 valence electrons in the neutral state with each Au atom contributing one electron to the valency. This corresponds to an electronic shell closing in the superatom complex model. The charge-state [2+] was selected to obtain a large HOMO-LUMO gap (0.56 eV) that occurs with hollow bilayer structures and a free-electron count of 90.[7] The degeneracy and level-ordering of the frontier orbitals also agrees well with the work of Chakraborty *et* al.[7]
(c) *I*–$Au_{60}(MgCp)_{12}$: this structure is composed by the *I*–Au_{60} shell with 12 stellating Magnesium atoms located at the 12 pentagons. Above each of the Mg ions is placed a cyclopentadienide (Cp) anion with five carbon and hydrogen atoms[8]. Once again, the *I*–symmetry and structure of the *I*–Au_{60} shell is kept after relaxation, its radius increases by 0.68%, while the Au–Au bond lengths of the pentagons increase by 1.11%, and 1.1% and 0% for the triangles. The HOMO-LUMO gap is calculated to be 1.07 eV. With respect to the electronic shell structure, Au atoms contribute one valence electron, Mg contributes two electrons, and each of the 12 Cp ligands withdraws one electron, resulting in a total of 72 valence electrons. This corresponds to a shell closing in hollow monolayer structures[9] resulting in an increased electronic stability shown by the opening up of the HOMO-LUMO gap of 1.07 eV.

Supplementary Note 3. In summary, our preliminary DFT calculations indicate that the *I*–Au_{60} shell is quite stable, for the *I*–symmetry is preserved and the shell is slightly deformed only for the case of I_h–C_{60}@*I*–Au_{60} system. Therefore the *I*–Au_{60} shell can be stabilized through different cores, as suggested in the main manuscript.

Supplementary Methods

Structural Optimizations and Vibrational Spectra

Spin-polarized density-functional theory (DFT) calculations were performed with the `SIESTA` code[10,11] to obtain the optimized structures. The generalized-gradient approximation (GGA) exchange–correlation functional of Perdew-Burke-Ernzerhof (PBE) was used.[12] The wave-functions were expanded in a double-ζ polarized basis set (DζP). Norm-conserving Troullier-Martins (TM) pseudopotentials (PPs)[13] with scalar relativistic correction were used, which include the d electrons in the valence, that is, with 11 valence electrons for each atom. After a thorough series of convergence tests, a 550 Rydberg cutoff for the density integration grid and a density matrix convergence criterion of 10^{-4} eV were chosen. All the Au atoms were allowed to relax using the conjugate gradient minimization method until the forces were smaller than 0.005 eV/Å. A simple cubic superlattice with a cell size of 40 Å was used. These parameters gave a bulk FCC cohesion energy of 3.20 eV/atom and a lattice constant of 4.18 Å (interatomic distance 2.95 Å), +2.4% larger than the experiment, which is typical of the GGA functional. The vibrational spectra of the C_1–Au_{60}, I–Au_{60}, and I–Au_{72} clusters were calculated within the same DFT-GGA-PBE approximation, a DζP basis set and TM PPs, using the VIBRA utility of the SIESTA code.[10,11]

I–Au_{72} structure

The I–Au_{72} structure shown in Fig. 1(g) in the main manuscript was first obtained using the same computational methodology followed by Karttunen *et al.*[9] These calculations were carried out using the GGA-BP86 functional and a def2-TZVP basis set (a polarized triple zeta basis set) employing a 19-valence-electron scalar-relativistic pseudopotential. Subsequently, when the I–Au_{72} was re-optimized using the SIESTA code with the parameters specified in this manuscript and then symmetrized with the Symmol tool,[14] the structure did not change significantly.

Structural Transition Tests from I_h–Au$_{60}$ to I–Au$_{60}$

The structural transition from the I_h–Au_{60} to the I–Au_{60} shell is remarkably robust. A series of thorough DFT convergence tests were performed to investigate and analyze the atomic structure and structural stability of the 60-atom Au shells. Specifically, two initial 60 –atom Au shell model structures were considered from the ubiquitous monolayer-protected 144-atom noble-metal cluster compound $Au_{144}(SR)_{60}$, with ligands -SR = -SCH_3 or -Cl in its neutral and charged state $[2^+, 4^+]$, respectively.[2–4]

Given the peculiar orderly contraction of the cluster, we also considered the uniform contraction of the atomic coordinates of I_h–Au_{60} by $\sim 10\%$, and the system underwent the same structural transition. The optimized coordinates from `SIESTA` were analyzed using the Symmol tool,[14] which confirmed the I– symmetry and returned a symmetrized version of the coordinates to remove random deviations from the fully relaxed geometry with zero forces on the atoms. The present work reports these symmetrized coordinates.

We have performed additional geometry optimizations of the I–Au_{60} with a higher level of theory, i.e., similar computational methodology and parameters as used by Karttunen *et al.*[9] Notwithstanding, the use of 19 valence electron pseudo-potential and a polarized triple zeta basis set also predicts the stability of the cage I–Au_{60} structure, with no big difference with respect to the SIESTA calculations.

Electronic structure calculations

The electronic density of states (eDOS) of the I–Au_{60}, I–Au_{72} and C_1–Au_{60} clusters shown in Fig. 3 of the manuscript were obtained from the octopus code[15,16] Kohn-Sham eigenvalues.

As an additional test, we have performed a B3LYP[17] calculation. This semi-empirical hybrid functional is known to work well for molecules and to do badly for bulk materials.[18] We have carried out a static ground-state calculation using the octopus code[15,16] and found that the electronic structure around the gap does not change strongly compared to the PBE calculation: E_g = 0.350 eV for B3LYP and 0.327 eV for PBE. Moreover, we have

carried out the projection of the occupied states on angular momentum states as presented in Fig. 3 of the original manuscript. Our result indicates clearly that the electronic structure does not change significantly between the B3LYP and PBE functionals, especially for the states around the gap. By contrast, the time-evolution turns out to be extremely heavy computationally when octopus is used with the B3LYP functional, making it impractical to do this within a reasonable time; furthermore, the discussion above holds also here and suggests that the quality of the PBE calculation is sufficient; the calculations using the GGA-PBE functional are accurate enough to support the results here presented.

Molecular Dynamics Calculations

We performed Born-Oppenheimer molecular dynamics simulations using the VASP code[19−21] with the PBE[12] functional at different temperatures: 100K, 150K, 200K, 250K, and 300K. We heated the structures before thermalizing for 2000 steps. Thereafter, the runs were restarted with random velocities to ensure that the history of the heating was "forgotten". Subsequently, we ran about 22 ps (30,000 steps, time step of 0.75 fs) in a microcanonical calculation (stability is monitored using the total energy, which is well-conserved over the long runs, up to a few meV). Our results indicate the existence of rather high energy barriers, which keep the I–Au_{60} in a metastable state, for the I–Au_{60} nanocage remains stable up to about 200K. Animations showing the structural development can be found in the Supplementary Movies 7 – 11.

TD-DFT Calculations

Absorption spectra are calculated using time-dependent density-functional theory (TD-DFT) as implemented in the real-space code octopus.[15,16] After a ground-state calculation, spectra are calculated using the time-evolution formalism.[22] Norm-conserving TM PPs and the GGA PBE were used. The real space grid spacing was set to 0.20 Å, the radius of the spheres centered around each atom which make up the calculation domain to 5 Å. The evolution

time was 25 fs and 250 fs, corresponding to a broadening of the spectra of about 0.15 eV and 0.015 eV. The time step for the propagation was 1.97×10^{-3} fs.

Supplementary References

1. Tran, N. T.; Powell, D. R.; Dahl, L. F. Nanosized $Pd_{145}(CO)_x(PEt_3)_{30}$ Containing a Capped Three-Shell 145-Atom Metal-Core Geometry of Pseudo Icosahedral Symmetry. *Angew. Chem. Int. Ed.* **39**, 4121-4125 (2000).

2. López-Acevedo, O.; Akola, J.; Whetten, R. L.; Grönbeck, H.; Häkkinen, H. Structure and Bonding in the Ubiquitous Icosahedral Metallic Gold Cluster $Au_{144}(SR)_{60}$. *J. Phys. Chem. C.* **113**, 5035-5038 (2009).

3. Bahena, D., et al. STEM Electron Diffraction and High-Resolution Images Used in the Determination of the Crystal Structure of the $Au_{144}(SR)_{60}$ Cluster. *J. Phys. Chem. Lett.* **4**, 975-981 (2013).

4. Tlahuice-Flores, A; Black, D. M.; Bach, S. B.; Jose-Yacamán, M.; Whetten, R. L. Structure & bonding of the gold-subhalide cluster *I*-$Au_{144}Cl_{60}$ [z]. *Phys. Chem. Chem. Phys.* **15**, 19191-19195 (2013).

5. Weissker; H.-C., et al. Information on quantum states pervades the visible spectrum of the ubiquitous $Au_{144}(SR)_{60}$ gold nanocluster. *Nat. Commun.* **5**, 3785 (2014).

6. Johansson, M. P.; Sundholm, D.; Vaara, J. Au_{32}: A 24-Carat Golden Fullerene. *Angew. Chem. Int. Ed.* **43**, 2678-2681 (2004).

7. Chakraborty, I., et al. The Superstable 25 kDa Monolayer Protected Silver Nanoparticle: Measurements and Interpretation as an Icosahedral $Ag_{152}(SCH_2CH_2Ph)_{60}$ Cluster. *Nano Lett.* **12**, 5861-5866 (2012).

8. Clayborne, P. A.; López-Acevedo, O.; Whetten, R. L.; Grönbeck, H.; Häkkinen, H.; The $Al_{50}Cp^*_{12}$ Cluster – A 138-Electron Closed Shell (L=6) Superatom. *Eur. J. Inorg. Chem.* **2011**, 2649-2652 (2011).

9. Karttunen, A. J.; Linnolahti, M.; Pakkanen, T. A.; Pyykko, P. Icosahedral Au_{72}: A Predicted Chrial and Spherically Aromatic Gold Fullerene. *Chem. Commun.* **4**, 465-467 (2008).

10. Soler, J. M., et al. Metallic bonding and cluster structure. *Phys. Rev. B*. **61**, 5771-5780 (2000).

11. Soler, J. M., et al. The SIESTA method for ab initio order-N materials simulation. *J. Phys. Condens. Matter*. **14**, 2745 (2002).

12. Perdew, J. P.; Burke, K.; Ernzerhof, M. Generalized Gradient Approximation Made Simple. *Phys. Rev. Lett.* **77**, 3865-3868 (1996).

13. Troullier, N.; Martins, J. L. Efficient pseudopotentials for plane-wave calculations. *Phys. Rev. B*. **43**, 1993-2006 (1991).

14. Pilati, T.; Forni, A. *SYMMOL* - a program to find the maximum symmetry in an atom cluster: an upgrade. *J. Appl. Crystallogr.* **33**, 417 (2000).

15. Marques, M. A. L.; Castro, A.; Bertsch, G. F.; Rubio, A. Octopus: a first-principles tool for excited electron-ion dynamics. *Comput. Phys. Commun.* **151**, 60-78 (2003).

16. Castro, A., et al. Octopus: a tool for the application of time-dependent density functional theory. *Phys. Status Solidi B*. **243**, 2465-2488 (2006).

17. Stephens, P. J.; Devlin, F. J.; Chabalowski, C. F.; Frisch, M. J. Ab Initio Calculation of Vibrational Absorption and Circular Dichroism Spectra Using Density Functional Force Fields. *J. Phys. Chem.* **98**, 11623-11627 (1994).

18. Ullrich, C. A.; Yang, Z.-H. A brief compendium of time-dependent density functional theory. *Braz. J. Phys.* **44**, 154-188 (2014).

19. Kresse, G.; Hafner, J. Ab initio molecular dynamics for liquid metals. *Phys. Rev. B*. **47**, 558 (1993).

20. Kresse, G.; Furthmüller, J. Efficiency of ab-initio total energy calculations for metals and semiconductors using a plane-wave basis set. *Comput. Mat. Sci.* **6**, 15 (1996).

21. Kresse, G.; Joubert, D. From ultrasoft pseudopotentials to the projector augmented wave method. *Phys. Rev. B*. **59**, 1758 (1999).

22. Yabana, K.; Bertsch, G. F. Time-dependent local-density approximation in real time. *Phys. Rev. B*. **54**, 4484-4487 (1996).
