# Supplementary material for: Chiral symmetry breaking yields the I-Au60 perfect golden shell of singular rigidity
Source: Nat Commun. 2018 Aug 22;9:3352. doi: 10.1038/s41467-018-05215-3 (PMC6105599; doi:10.1038/s41467-018-05215-3)
Supplement: Supplementary file 3 — Description of Additional Supplementary Information [file 41467_2018_5215_MOESM3_ESM.pdf]

Description of Additional Supplementary Files

File Name: Supplementary Movie 1
Description: Animation of the structural relaxation of the I_h–Au_{60} into the I–Au_{60}. The structure first undergoes a uniform contraction followed by a rotation of the pentagons associated with the transition from the I_h–Au_{60} rhombicosidodecahedron (3.4.5.4) to the I–Au_{60} snub dodecahedron (34 .5*) morphology. Select atoms and bonds are colored to highlight the rotation of the triangles (red) and pentagons (blue).

File Name: Supplementary Movie 2
Description: Animation of vibrational mode 1176, corresponds to combinations of symmetrical and asymmetrical stretching modes.

File Name: Supplementary Movie 3
Description: Animation of vibrational mode 1177, corresponds to combinations of symmetrical and asymmetrical stretching modes.

File Name: Supplementary Movie 4
Description: Animation of vibrational mode 1178, corresponds to combinations of symmetrical and asymmetrical stretching modes.

File Name: Supplementary Movie 5
Description: Animation of vibrational mode 1179, corresponds to combinations of symmetrical and asymmetrical stretching modes.

File Name: Supplementary Movie 6
Description: Animation of vibrational mode 1180, corresponds to combinations of symmetrical and asymmetrical stretching modes

File Name: Supplementary Movie 7
Description: Animation of the molecular dynamics simulation at 100K. The MD simulation shows that at 100K the I–Au_{60} cluster retains its cage structure. The temperature is basically constant, i.e., there is no drift or systematic increase of the temperature.

File Name: Supplementary Movie 8
Description: Animation of the molecular dynamics simulation at 150K. The MD simulation shows that at 150K the I–Au_{60} cluster retains its cage structure. The temperature is basically constant, i.e., there is no drift or systematic increase of the temperature.

File Name: Supplementary Movie 9
Description: Animation of the molecular dynamics simulation at 200K. The MD simulation shows that at 200K the I–Au_{60} cluster retains its cage structure. The temperature is basically constant, i.e., there is no drift or systematic increase of the temperature.

File Name: Supplementary Movie 10
Description: Animation of the molecular dynamics simulation at 250K. The temperature rises significantly after about 10 ps. This indicates that the structure has started to move into a lower-energy configuration, changing the connectivity and symmetry.

File Name: Supplementary Movie 11
Description: Animation of the molecular dynamics simulation at 300K. The temperature rises significantly after about 5 ps. This indicates that the structure has started to move into a lower-energy configuration, changing the connectivity and symmetry.

File Name: Supplementary Data 1
Description: XYZ file of I_h–Au_{60} structure

File Name: Supplementary Data 2
Description: XYZ file of I–Au_{60} structure.

File Name: Supplementary Data 3
Description: XYZ file of I_h–Au_{72} structure

File Name: Supplementary Data 4
Description: XYZ file of C_1–Au_{60} structure.

File Name: Supplementary Data 5
Description: Table of vibrational frequencies for the C_1–Au_{60}, I–Au_{60}, and I_h–Au_{72}.
